# Synthesis of high drug loading, reactive oxygen species and esterase dual-responsive polymeric micelles for drug delivery†

**DOI:** 10.1039/c8ra09770d

**Published:** 2019-01-18

**Authors:** Nan Wang, Xiao-Chuan Chen, Ruo-Lin Ding, Xian-Ling Yang, Jun Li, Xiao-Qi Yu, Kun Li, Xi Wei

**Affiliations:** Key Laboratory of Green Chemistry and Technology, Ministry of Education, College of Chemistry, Sichuan University Chengdu China 610064 xqyu@scu.edu.cn; Operative Dentistry and Endodontics, Guanghua School of Stomatology, Affiliated Stomatological Hospital, Guangdong Province Key Laboratory of Stomatology, Sun Yat-sen University Guangzhou Guangdong China 510055 weixi@mail.sysu.edu.cn; West China College of Stomatology, Sichuan University Chengdu China 610064

## Abstract

Stimulus-responsive, controlled-release systems are of great importance in medical science and have drawn significant research attention, leading to the development of many stimulus-responsive materials over the past few decades. However, these materials are mainly designed to respond to external stimuli and ignore the key problem of the amount of drug loading. In this study, exploiting the synergistic effect of boronic esters and *N*-isopropylacrylamide (NIPAM) pendant, we present a copolymer as an ROS and esterase dual-stimulus responsive drug delivery system that has a drug loading of up to 6.99 wt% and an entrapment efficiency of 76.9%. This copolymer can successfully self-assemble into polymer micelles in water with a narrow distribution. Additionally, the measured CMC hinted at the good stability of the polymeric micelles in water solution, ensuing long circulation time in the body. This strategy for increasing the drug loading on the basis of stimulus response opens up a new avenue for the development of drug delivery systems.

## Abstract 2

A novel high drug loading, controlled-release drug delivery system was constructed with dual-stimulus responsive abilities in cells.

## Graphical abstract

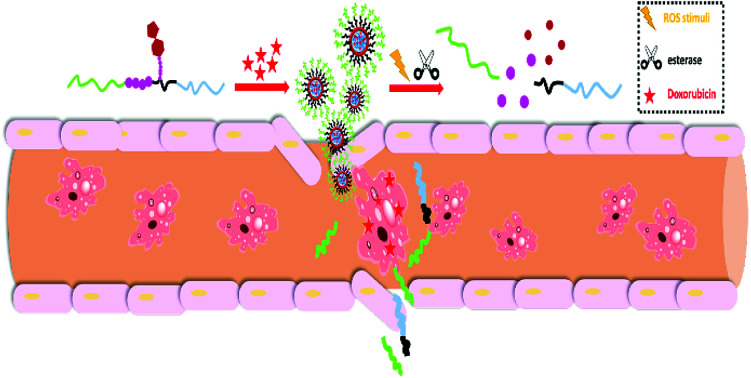

## Introduction

In recent decades, multifunctional polymeric materials have attracted growing interest due to their wide range of applications, especially in the biomedical field.^1^ These polymeric materials achieve better control of architecture^2^ and functionality *via* the copolymerization of different monomers. Among the numerous polymerization methods, reversible addition-fragmentation chain transfer (RAFT) polymerization has proven to be an excellent and convenient route to block co-polymers *via* the use of the desired RAFT agents.^3^ Meanwhile, by selectively incorporating certain functional groups, desired amphiphilic block copolymers or responsive materials can be synthesized using RAFT methods. In aqueous solutions, amphiphilic block copolymers can self-assemble into a variety of different nanostructures, including spherical micelles, cylindrical micelles, and drug-delivery vesicles.^4^ These smart materials show mono-, dual- or multi-responsive abilities and can efficiently response to external stimuli.^5^ For example, Qin *et al.* prepared a boronic acid block copolymer whose morphology is controlled by the regulation of the pH.^6^ Sumerlin *et al.* prepared pH- or sugar-responsive block copolymers *via* RAFT polymerization.^7^ Sumerlin's group also combined pH- and sugar-responsiveness with the thermo-responsiveness of NIPAM to prepare block copolymers.^1^ However, biological applications have not been explored in their studies. And according to literature, esterase are expressed in numerous tissues.^8^ And a series of esterase-responsive probes have been designed.^9^ In 2016, Shen's group taking advantage of the high esterase activity in HeLa cancer cells to design a esterase-responsive charge-reversal polymer, whose polyplexes had a selective gene expression in the cancer cells high in esterases.^10^ Generally, although pH, glucose-sensitive or multiple-sensitivity boronic acid block polymers have already been reported, high drug loading, ROS and esterase-responsive materials for drug delivery have not been synthesized or investigated to date.

**Scheme 1:**
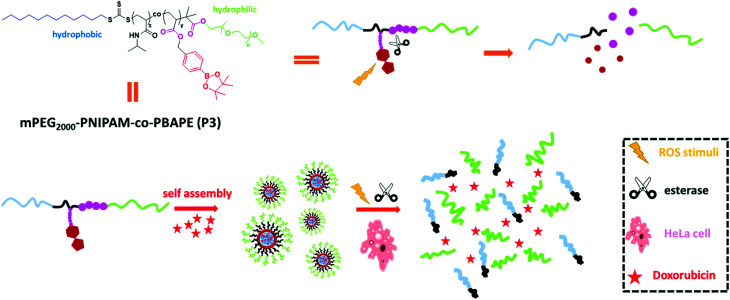
Self-assembly properties and stimuli-responsive release properties of mPEG_2000_-PNIPAM-*co*-PBAPE (P3).

Herein, we constructed a di-block copolymer system containing boronic esters and *N*-isopropyl acrylamide *via* RAFT methods that offers genuine synergies between the esterase and ROS stimuli. Arylboronic esters could be oxidized by ROS (*e.g.*, H_2_O_2_)^11^ and then undergo rearrangement to unmask the modified group, and the ester bonds could be hydrolysed by esterase.^12^ Hydrogen peroxide (H_2_O_2_) is one of the most important ROS and plays critical roles in a number of physiological and pathological processes.^13^ This reactive strategy has been usually applied to design small-molecule-based fluorescent probes in recent years.^14^ In addition to dual-stimulus responsiveness, phenylboronic acid, pinacol ester pendent can function as an electron acceptor that affords donor–acceptor coordination with doxorubicin to obtain micelles with a high drug-loading ability.^15^ This kind of co-polymer can self-assemble into novel polymeric micelles that show low cytotoxicity, low critical micelle concentration, high drug-loading and dual response to external stimuli. Moreover, *in vitro* drug-release studies revealed that this polymeric system can serve as a novel controlled-release system (Scheme 1).

## Experimental

### Materials and methods

Acryloyl chloride (96%), carbon disulphide (AR), 1-dodecanethiol (99.5%), methyl-trioctylammonium chloride (97%), poly(ethylene glycol) (*M*_n_ ∼ 2000), triethylamine (>99%), *N*,*N*′-dicyclohexyl carbodiimide (99%), 4-(dimethyl amino)-pyridine (99%), *N*-isopropylacrylamide (98%), doxorubicin hydrochloride (DOX-HCl) (98%), and 2,2′-azobis(2-methyl-propionitrile) (98%), were purchased from Aladdin Reagent (Shanghai, China), and esterase was purchased from Sigma Aldrich. Sodium hydroxide, 4-hydroxymethylphenylboronic acid, and pinacol ester (98%) were purchased from Energy Chemical Reagent (Shanghai, China). The *N*-isopropylacrylamide was recrystallized from *n*-hexane three times prior to use, and 2,2′-azobis(2-methylpropionitrile) was recrystallized from ethanol three times to afford a white acicular crystal. Dichloromethane (DCM), 1,4-dioxane, acetone, and trichloromethane (CHCl_3_) were dried and distilled first with purification. HeLa and HL-7702 cells were obtained from Shanghai Institute of Biochemistry and Cell Biochemistry and Cell Biology, Chinese Academy of Science. Dulbecco's modified Eagle's medium (DMEM), Roswell Park Memorial Institute medium 1640 (RPMI 1640), 3-(4,5-dimethylthiazol-2-yl)-5-(3-carboxymethoxyphenyl)-2-(4-sulphophenyl)-2*H* tetrazolium (MTS), and foetal bovine serum (FBS) were provided by Gibco (Lige Technologies, Switzerland). Anti-HER2 Affibody was purchased from Affibody (Solna, Sweden).

^1^H NMR and ^13^C NMR spectra were measured using a Bruker AM 400 NMR spectrometer. Proton chemical shifts of the NMR spectra were given in ppm relative to the internal TMS reference (^1^H, 0.00 ppm). ESI-HRMS spectral data were recorded using a Finnigan LCQDECA mass spectrometer. Fluorescence emission spectra were obtained using a Hitachi F-7000 spectrometer at 298 K. Gel permeation chromatography (GPC) was carried to confirm the polymer molecular weights and their distributions using a Waters HPLC system equipped with a model 1515 isocratic pump, a 717 plus autosampler, and a 2424 refractive index (RI) detector with Waters Styragel® HT3 and HT4 columns in series. The eluting solvent was THF at a flow rate of 1.0 mL min^−1^ at 45 °C. The retention times were calibrated against a poly(ethylene glycol) standard with a molecular weight range of 600–80 000 Da. Transmission electron microscopy (TEM, Hitachi H-600) at an acceleration voltage of 100 kV was performed to investigate the micelle morphology. Dynamic light scattering (DLS) measurements were carried out at 25 °C using a Zetasizer Nano-ZS90 system from Malvern Instruments equipped with a 633 nm HeNe laser using backscattering detection with a fixed detector angle of 90°. Confocal lasing scanning microscopic (CLSM) images of single-photo were obtained using LSM 780 (Zeiss) and Nikon A1R MP^+^, and FTIR spectra were recorded using a Shimadzu FTIR-4200 spectrometer. Ultrapure grade 10× phosphate-buffered saline (PBS) buffer with pH = 7.4 was purchased from first BASE Singapore. Milli-Q water was supplied by Milli-Q Plus System (Millipore Corporation, Bedford, MA).

### Synthetic procedures and characterization

#### Synthesis of the CTA-COOH

First, a small-molecule-based RAFT agent was synthesized according to the reported method.^16^ Generally, a three-neck flask was cooled below 10 °C with a cold circulating pump while removing the air contained in the flask with nitrogen. Then, 1-dodecanethiol (12.11 g, 59.85 mmol), acetone (28.86 g, 496.90 mmol), and Aliquot 336 (tricaprylylmethylammonium chloride, 0.97 g, 2.41 mmol) were added, followed by the dropwise addition of a sodium hydroxide solution (50 wt%, 5.03 g). The reaction mixture was stirred for another 15 min before carbon disulphide (4.563 g, 60 mmol) in acetone (6.054 g, 103.5 mmol) was added, and during this time, the mixture turned red. Ten minutes later, chloroform (10.688 g, 90 mmol) was added in one portion, followed by the dropwise addition of a 50% sodium hydroxide solution (24 g, 300 mmol) over 30 min. The reaction mixture was stirred overnight, and 90 mL of water was added, followed by 15 mL of concentrated HCl to acidify the aqueous solution. Nitrogen gas was purged through the reactor with vigorous stirring to assist the evaporation of acetone. The solid was collected through filtering and then was dissolved in 150 mL of 2-propanol. The undissolved solid was filtered off, and the rest of the 2-propanol solution was concentrated and dried, and the resulting solid was recrystallized from *n*-hexane to obtain 12.4 g of yellow crystalline solid (yield: 57%). ^1^H NMR (400 MHz, CDCl_3_) *δ* 3.31–3.25 (t, 2H), 1.72 (s, 6H), 1.70–1.63 (m, 2H), 1.25 (m, 18H), 0.88 (t, *J* = 6.9 Hz, 3H). ^13^C NMR (101 MHz, CDCl_3_) *δ* 220.8, 178.4, 55.5, 37.0, 31.9, 29.6, 29.5, 29.4, 29.3, 29.1, 29.0, 27.8, 25.2, 22.7, 14.1. HRMS (ESI): *m*/*z*: calcd for C_17_H_32_O_2_S_3_Na: 387.1462 [M + Na]^+^; found: 387.1460.

#### Synthesis of the marco-CTA-COOH (P1)

The CTA-COOH was conjugated on the pendant of mPEG_2000_-OH through an esterification reaction.^17^ Briefly, mPEG_2000_-OH (1.9397 mmol) and DMAP (1.4218 g, 11.6382 mmol) were dissolved in anhydrous DCM (20 mL) and placed in a 50 mL round-bottom flask in an ice bath, followed by the addition of DCC (0.2397 g, 1.1638 mmol) with a magnetic stirring bar, and CTA-COOH (0.7073 g, 1.9397 mmol) was dissolved into anhydrous DCM and dropped slowly into the flask. The reaction was carried out for 2 h in an ice bath and allowed to reach room temperature for another 24 h with stirring. Then, the white precipitate was filtered out, and the remaining filtrate of the P1 was precipitated in cold diethyl ether (40 mL) twice in order to remove the unreacted small molecule compounds. After purification and lyophilisation, the macro-CTA-COOH (P1) was obtained as a light yellow solid (4.10 g). ^1^H NMR (400 MHz, CDCl_3_) *δ* 4.28–4.21 (t, 2H), 3.28–3.21 (t, 2H), 1.69 (s, 6H), 1.24 (m, 18H), 0.88 (t, *J* = 11.6, 5.0 Hz, 3H. Additionally, the synthesis of P1 was also confirmed by matrix-assisted laser desorption ionization time-of-flight mass spectrometry (MALDI-TOF MS), and the matrix was prepared at a concentration of 10 mg mL^−1^.

#### Synthesis of the mPEG_2000_-PNIPAM (P2)

The polymer mPEG_2000_-PNIPAM (P2) was synthesized by reversible addition-fragmentation chain transfer polymerization.^18^ Briefly, *N*-isopropylacrylamide (1.107 g, 9.78 mmol) was initially dissolved in 20 mL of 1,4-dixone in a Schlenk tube under ultrasonication, followed by dissolving macro-CTA-COOH (1.2 g, 0.51 mmol), and AIBN (2 mg) into this mixture. After three freeze–pump–thaw cycles, the polymerization was allowed to proceed at 70 °C for 24 hours. Then, the polymerization was quenched by cooling the system, and the resulting solution was precipitated with excess amount of cold diethyl ether three times. The precipitated copolymer was collected by filtration and was subjected to dialysis against ultrapure water for 48 h. A white powder was finally obtained with a yield of 2.04 g after freeze drying.

#### Synthesis of the B1-AC

Acrylate-ended B1 (B1-AC) was synthesized according to the references with a few modifications.^19^ In brief, 4-hydroxymethylphenylboronic acid, pinacol ester monomer (7.39 mmol) was dissolved into anhydrous DCM at 20% solids concentration in a Schlenk flask. TEA (11.09 mmol, 1.5 eq.) was added with gentle stirring, and the flask was immersed in an ice bath, followed by the dropwise addition of acryloyl chloride (11.09 mmol, 1.5 eq.) for 10 min. The reaction was carried out for 2 h in an ice bath, and the reaction mixture was allowed to reach room temperature for further 24 h with stirring. After washing with dilute HCl solution three times and precipitating, the solids were dried under vacuum for 48 h, and then the crude product was further purified by column chromatography by using ethyl acetate/petroleum ether (1 : 20) as the eluent to afford a white solid (1.428 g, 67%).^1^H NMR (400 MHz, CDCl_3_) *δ* 7.81 (d, *J* = 8.0 Hz, 2H), 7.38 (d, *J* = 8.0 Hz, 2H), 6.45 (dd, *J* = 17.3, 1.4 Hz, 1H), 6.17 (dd, *J* = 17.3, 10.4 Hz, 1H), 5.86 (dd, *J* = 10.4, 1.4 Hz, 1H), 5.21 (s, 2H), 1.34 (s, 12H). ^13^C NMR (101 MHz, CDCl_3_) *δ* 166.1, 139.0, 135.1, 131.3, 128.4, 127.4, 84.0, 66.3, 25.0. HRMS (ESI): *m*/*z*: calcd for C_16_H_21_BO_4_ Na: 311.1431 [M + Na]^+^; found: 311.1426.

#### Synthesis of the mPEG_2000_-PNIPAM-*co*-PBAPE (P3)

The synthesis steps for obtaining P3 were very similar to those used to obtain P2. Briefly, B1-AC (1.107 g, 9.78 mmol) was initially dissolved in 1,4-dixone (20 mL) in a Schlenk tube under ultrasonication, followed by dissolving P2 (1.2 g, 0.51 mmol), and AIBN (2 mg) into this mixture. After three freeze–pump–thaw cycles, the polymerization was allowed to proceed at 70 °C for 24 hours. Then, the polymerization was quenched by cooling the system, and the resulting solution was precipitated with excess cold diethyl ether three times. The precipitated copolymer was collected by filtration and was subjected to dialysis against ultrapure water for 48 h. A white powder was finally obtained with a yield of 1.04 g after freeze drying.

#### IR-characterization

The chemical structures of the as-synthesized P2 and P3 were further analysed using a Shimadzu FTIR-4200 spectrometer.

### Preparation of polymeric micelles

#### Preparation of polymeric micelles P3

Micelles of P3 were prepared using the dialysis method. The polymers (30 mg) were dissolved in 6 mL DMF. Then, 5 mL deionized water was added to the solution under gentle stirring. After stirring for 2 h at room temperature, the solution was transferred into a dialysis membrane (MWCO 2000 Da) and dialyzed for 48 h against deionized water. The outer phase was replaced with fresh deionized water every 6 h. The resulting solution was finally lyophilized. The preparation method of micelles P2 is very similar to that for P3, and specific experimental steps are shown in ESI.†

#### Preparation of DOX-loaded polymeric micelles P3

Here, we identified a common anticancer drug, doxorubicin (DOX), as a model drug, and the water-insoluble DOX was encapsulated into P3 micelles by adding 1.5 mL of 2 mg mL^−1^ DOX in DMF to 6 mL of P3 solution in DMF (5 mg mL^−1^), followed by stirring at room temperature for two hours in absolute darkness condition to stabilize the system, and then, the resulting solution was placed in a dialysis membrane (MWCO 2000 Da) and dialyzed for 48 h against deionized water, with constant stirring and frequent water changes. Then, the solution was filtered through a 0.45 μm syringe filter, and dynamic light scattering (DLS) measurements were conducted at 25 °C. The preparation method of DOX-loaded polymeric micelles P2 is very similar to that for P3, and specific experimental steps are shown in ESI.†

### Critical micelle concentrations determination

The critical micelle concentration (CMC) was determined using Nile Red as the fluorescence probe. The block copolymer concentration varied from 1.0 × 10^−6^ mg mL^−1^ to 0.2 mg mL^−1^, and the Nile Red concentration was fixed at 1.0 × 10^−6^ M. The fluorescence spectra were recorded using a Hitachi F-7000 spectrometer at 298 K. Both the emission and excitation slit widths were 5 nm. The samples were excited at 560 nm, and the emission spectra were recorded from 580 to 800 nm. The emission fluorescence values, *I*_631.6_ at 631.6 nm was used for the subsequent calculations. The CMC was determined from the plots of the *I*_631.6_*versus* the logarithm of the polymer concentration using the intersection of the linear regression lines as the CMC value.

### TEM and DLS characterization

The micelle solutions of P3 and DOX-loaded P3 at a concentration of 0.5 mg mL^−1^ were prepared as described above. To further study the formation of the micelles, their morphologies and sizes were also characterized by TEM and DLS measurements.

#### Entrapment efficiency and drug loading capacity determination

The standard curve for the determination of the free DOX content was measured by the fluorescence method with a Hitachi F-7000 spectrometer. The concentration of free DOX was calculated by the standard curve (Fig. S8†). The encapsulation efficiency and loading of free DOX were obtained by the following formulas.^20^1
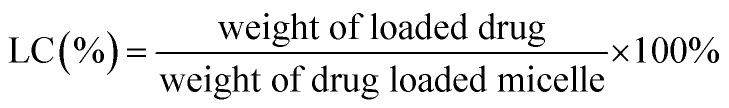
2
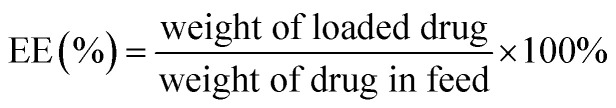


#### *In vitro* drug release experiments

The release test of the polymeric micelle nanocarrier platform was determined by the dialysis method (MW: cut-off: 3.5–5 kDa) at 37 °C. In short, dialysis membrane carrying 2.0 mL of DOX-loaded polymeric micelle (P3/DOX) were immersed in 20 mL of PBS solution (0 mM H_2_O_2_, 10 mM H_2_O_2_, 10 mM H_2_O_2_ and 30 μg mL^−1^ esterase, 0.2 mg mL^−1^) in a conical flask in the shaking incubator with the stirring speed of 100 rpm while the bulk polymeric micelle immersed in the PBS solution (0 mM H_2_O_2_ and no esterase, 0.2 mg mL^−1^) under the same conditions were used as control groups. At predetermined intervals, aliquots of media (1.0 mL) were taken and quantified by measuring at 500 nm with the Hitachi F-7000 spectrometer. Throughout the entire process, the total volume was kept constant.

### *In vitro* drug release experiments

HeLa cells (or HL-7702 cells) were cultured in Dulbecco's modified Eagle medium (DMEM) (or in Roswell Park Memorial Institute (RPMI) 1640 Medium (1640)) containing 10% foetal bovine serum and 1% antibiotic–antimycotic (penicillin–streptomycin, 10 000 U mL^−1^) at 37 °C in a 5% CO_2_/95% air incubator, and the medium was replenished every other day.

### Cell imaging experiments

For fluorescence imaging, cells (4 × 10^3^/well) were passed on a 6-well plate and incubated for 48 h. Prior to the staining experiment, the cells were washed twice with physiological saline, incubated with 14 mg L^−1^ DOX-loaded polymeric micelle P3/DOX for different times (2 h, 4 h, 8 h, 12 h, 24 and 48 h) at 37 °C, and then were washed twice with physiological saline. The confocal fluorescent images were captured with an excitation light at 488 nm. At the same time, DOX and DOX-HCl under the same conditions were used as control groups (1 mg L^−1^).

### *In vitro* cytotoxicity assay

The materials' toxicity towards HeLa cells was determined by MTS Cell Proliferation Colorimetric Assay Kit following the procedures described in the literature. Approximately 104 cells per well were seeded in 96-well plates and cultured overnight for 70–80% cell confluence. The medium was replaced with 100 μL of fresh medium with different concentration of the materials, to which 100 μL complexes were added at 200 μL. After 48 hours, 100 μL of 20% MTS solution in PBS was replaced with the old medium in each well for additional 0.5 h incubation. The metabolic activity of the probe-treated cells was expressed relative to untreated cell controls taken as 100% metabolic activity. The cytotoxicity study towards HL-7702 cells was carried out following the same procedure.

## Results and discussion

### Synthesis and characterization

P1 was synthesized *via* esterification. As shown in Fig. S1,† the reversible addition-fragmentation chain transfer (RAFT) agent CTA-COOH was grafted with mPEG_2000_-OH by the esterification reaction using DCC and DMAP as the coupling agent and catalyst, respectively. Fig. S2† shows the matrix-assisted laser desorption ionization time-of-flight mass spectrum (MALDI-TOF MS) of P1. The MALDI-TOF spectrum of P1 has systematic peaks centred at ∼1990 *m*/*z*, extending from 1400 to 2500 *m*/*z*. The difference in the *m*/*z* values between the neighbouring peaks is 44.036, corresponding to the molar mass of the mPEG_2000_ unit. Then, the monoblock polymer P2 and diblock polymer P3 were successfully synthesized by using the macromolecular chain transfer reagent P1. P2, and P3 were synthesized and detected, as shown in Table 1. We can also calculate the degree of polymerization of polymer P2 and P3 according to the results of NMR. Fig. S3† shows the particle size distributions of P2 micelles and we carried out the thermal responsive properties of materials P2 as it shown in Fig. S4 and S5† shows the macro change of P2 temperature sensitivity. In addition, Fig. S6† also examines the temperature-sensitive properties of P3. In the present study, P1 was grafted as the water-soluble part by conjugating it to the *N*-isopropylacrylamide segment, and then the polymer was also modified by boronic acid, pinacol ester on the side chain introduced to obtain ROS sensitivity and increase the drug loading.

**Table 1:** Basic characterization of the polymeric micelles

Sample	*M*_n_ a	*M*_w_ a	*M*_z_ a	*M*_w_/*M*_n_^a^	Size^b^ (nm)	PDI^b^
P2	7393	8768	10 436	1.19	148.6 ± 0.36	0.135 ± 0.01
P3	8220	10 641	13 623	1.29	64.84 ± 0.22	0.154 ± 0.001

a Determined by GPC against a poly(ethylene glycol) standard.

b The size and PdI (polydispersity index) of the micelles were determined by DLS.

### IR-characterization

**Fig. 1:**
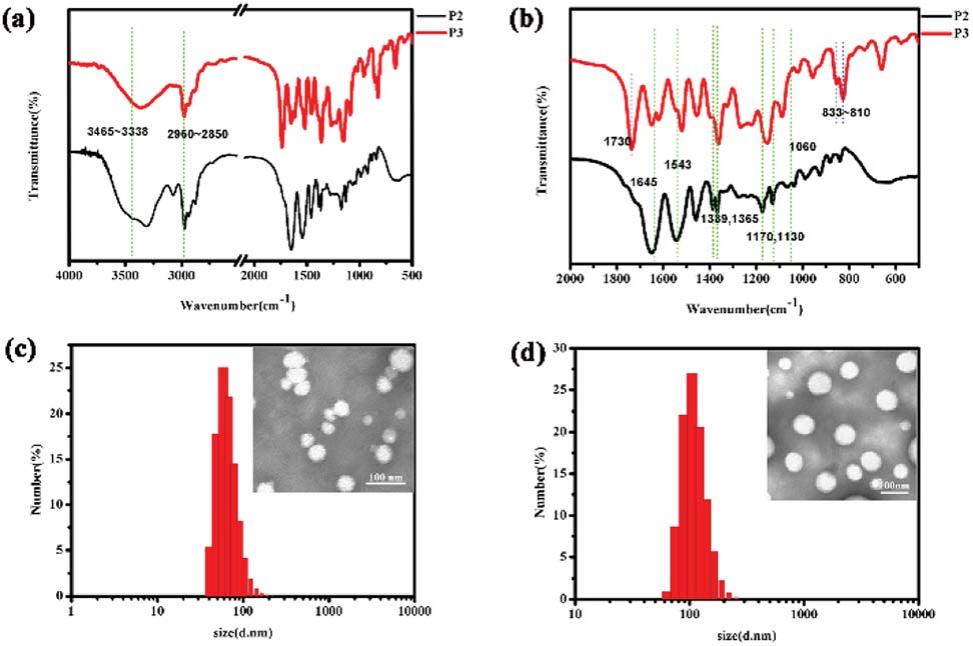
The IR spectrum of mPEG_2000_-PNIPAM (P2) and mPEG_2000_-PNIPAM-*co*-PBAPE (P3) (a and b). TEM images and particle size distributions of P3 (c) and DOX-loaded P3 (d).

As shown in Fig. 1, in the P2 spectrum, the peaks at 3465, 3420, and 3338 cm^−1^ correspond to the N–H stretching vibration of the –NH– group, whereas the weak band at 3035 cm^−1^ is due to the aromatic C–H stretching vibration, the peaks at 2960–2850 cm^−1^ correspond to the alkane C–H vibration, the band at 1645 cm^−1^ is due to the C=O stretching vibration in amide I bonds, and the peaks at 1543 cm^−1^ can be ascribed to the N–H stretching vibrations of the amide II bonds. The peaks at 1389 and 1365 cm^−1^ are induced by the deformed vibration absorption peak of the two methyl in –CH (CH3)_2_–, while the two peaks at 1170 and 1130 cm^−1^ are associated with the C–O stretching mode in the ester and the absorption peak of the PEG ether bond at 1060 cm^−1^ covers the C=S bond absorption. These characteristic peaks prove that the *N*-isopropylacrylamide monomer was successfully polymerized onto the polymer chain. Additionally, in the P3 spectrum, the strong peaks at 833–810 cm^−1^ are derived from 1,4-two substituted benzene, while the peak at 1730 cm^−1^ is ascribe to the overtone bands.

### Characterization of polymeric micelles P3 and DOX-loaded P3

Because the amphiphilic polymer P3 can self-assemble into micelles in aqueous solutions, to the best of our knowledge, the micelles can self-assemble above a certain threshold concentration, leading to a rapid increase in the emission intensity of Nile Red, as shown in Fig. S7.† The measured CMC values hinted at the good stability of these polymeric micelles in solution owing to their low CMC values, even after extreme dilution by the larger volume of systemic circulation *in vivo*.

Furthermore, as shown in Fig. 1(c and d), TEM characterization reveals that most of the polymer particles were spherical, further supporting the conclusion that polymeric micelles were formed. The results of the DLS measurements are also presented in Table 1. The results showed that the P3 spherical micelles had average diameters of 60–70 nm whereas the DOX-loaded P3 had the average diameters of 100–110 nm. These observations provided clear evidence not only for the micellization of P3 in water, similar to the results of the CMC analysis did, but also for their rather uniform morphology and size.

### Entrapment efficiency and drug loading capacity determination

The entrapment efficiency and loading capacity of free DOX of P3 were calculated by the standard curve, according to the following equation. Entrapment efficiency = 76.9%; loading capacity = 6.99 wt%. We also investigated the entrapment efficiency and loading capacity of P2, and the data were shown in Fig. S9.†

### *In vitro* release of DOX from prodrug DOX-loaded P3

**Fig. 2:**
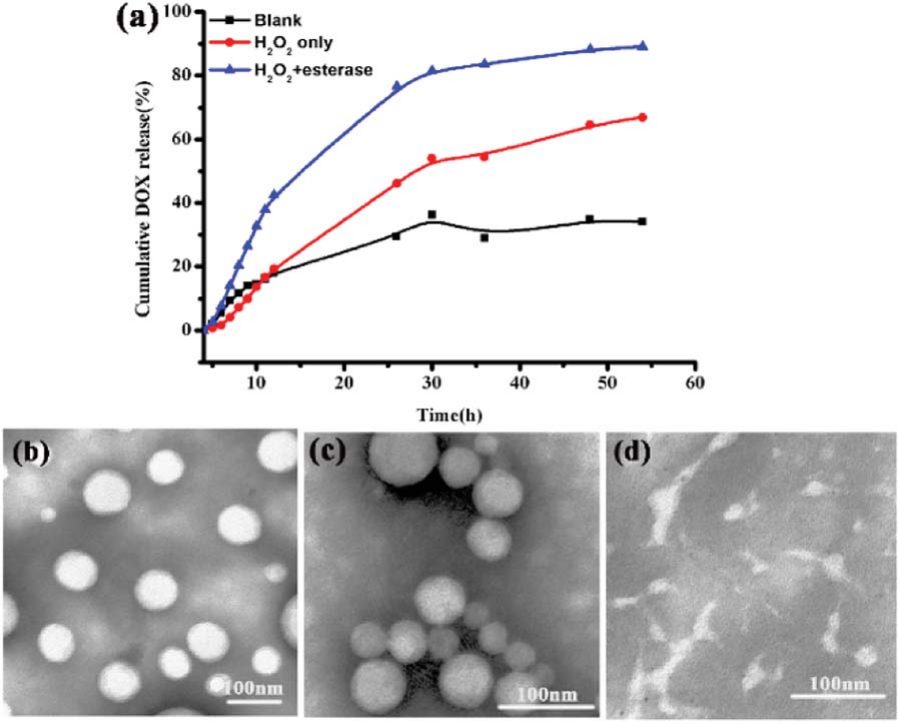
(a) Release profiles of DOX from P3 micelles at different conditions (black: blank; red: 10 mM H_2_O_2_; blue: 10 mM H_2_O_2_ + 30 μg mL^−1^ esterase). (b–d) TEM images of DOX-loaded P3, DOX-loaded P3 with 10 mM H_2_O_2_; DOX-loaded P3 with 10 mM H_2_O_2_ and 30 μg mL^−1^ esterase.

*In vitro* drug release performances of the DOX-loaded P3 under physiological conditions (PBS, pH 7.4, control), oxidation environment only (PBS, 10 mM H_2_O_2_) and both oxidation and esterase (PBS, 10 mM H_2_O_2_ with 30 μg mL^−1^ esterase) conditions were investigated, as shown in Fig. 2. The drug release rate was significantly influenced by both H_2_O_2_ and esterase, providing an additional indication of the dual-stimulus-sensitivity of DOX-loaded P3. At blank conditions, the prodrug DOX-loaded P3 show weak release, and the cumulative release amount of DOX was only 30% for 54 h, resulting from the stable and compact DOX-loaded P3 at normal physiological conditions. In 10 mM H_2_O_2_, the cumulated release of 54 h drugs was 30% higher than that in the blank group, as shown in Fig. 2(a), and the TEM image of the release fluid presented in Fig. 2(c) shows that these micelles were adhesive and some particles are smaller than those observed in Fig. 2(b). In contrast, in the presence of both H_2_O_2_ and esterase, the drug release rate was accelerated obviously, and the cumulative release amount reached 90%, and as shown in Fig. 2(a), these micelles were completely destroyed as observed from the TEM image presented in Fig. 2(d). Furthermore, as shown in Fig. S10 and Table S1,† we investigated the particle size changes of P3 under different *in vitro* conditions. Compared to P3, the size of the particles increased with the addition of H_2_O_2_ and the addition of both esterase and H_2_O_2_. Meanwhile, we investigated the *in vitro* release in the presence of different esterase concentrations, and as shown in Fig. S11(a),† it was found that compared to the control group, the amount of the release did not increase significantly; this is in line with the TEM observations shown in Fig. S11(b).† This may be due be to the rearrangement of ROS-sensitive DOX-loaded P3 pendant and the cleavage of the esterase-sensitive main chain on the copolymer.

### *In vitro* cellular uptake of DOX

**Fig. 3:**
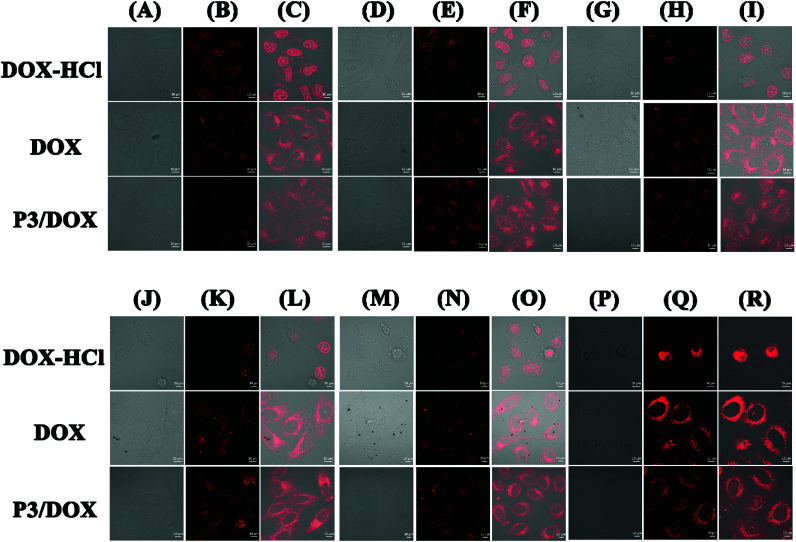
Cell imaging (14 μg mL^−1^) in HeLa cells: P3/DOX (A)-P3/DOX (R); (1 μg mL^−1^) in HeLa cells: DOX-HCl (A)-DOX-HCl (R); (1 μg mL^−1^) in HeLa cells: DOX (A)-DOX (R); column (A, D, G, J, M, P) bright field, column (B, E, H, K, N, Q) 488 nm excitations, column (C, F, I, L, O, R) merged image, column (A, B, C) 2 h, column (D, E, F) 4 h, column (G, H, I) 8 h, column (J, K, L) 12 h, column (M, N, O) 24 h, column (P, Q, R) 48 h, (scale bar = 10 μm).

The intracellular uptake of DOX-HCl, free DOX and DOX-loaded P3 was investigated using confocal lasing scanning microscopy (CLSM) (Fig. 3). When the cells were incubated with DOX-HCl, the cellular uptake of DOX-HCl was the same, regardless of the different time duration of 2, 4, 8, 12, 24 and 48 h. DOX-HCl was distributed in the nuclear region within 2 h of incubation, and strong fluorescence was observed in the nucleus, as reported previously.^21^ From column DOX-HCl (P)-DOX-HCl (R), after 48 h incubation, it was clearly observed that the HeLa cell morphology was completely destroyed. However, when the cells were incubated with free DOX, the cellular uptake of free DOX was also the same, regardless of different time durations of 2 h, 4 h, 8 h, 12 h, 24 h and 48 h, and free DOX was distributed in the cytoplasm region during long-time incubation, with the aggregation of free DOX with increasing time. Moreover, it was difficult to for the free DOX to transfer into the nucleus even after 48 h. When the cells were incubated with DOX-loaded polymeric micelle P3 in two hours, considerable fluorescence intensity was detected mainly in the cytoplasm, suggesting novel cellular uptake ability of DOX-loaded polymeric micelle P3, and with the prolongation of the incubation time, the cellular fluorescence was much higher. We found that after 4 hours, the DOX molecules were localized in the cell nuclei, and as expected from the drug release *in vitro* experiment, DOX localized in the cell nuclei is likely to be intercalated into DNA strands, thereby showing its toxicity against tumour cells. This dual cellular uptake profile of DOX molecules is likely due to the rapid demicellization of the DOX-loaded polymeric micelles under cellular conditions, leading to the rapid release of DOX from polymeric micelles and its subsequent internalization into cell nuclei. As shown in Fig. S12,† the average fluorescence intensity in the nucleus is a measure of the amount of DOX that enters the nucleus. When the cells were incubated with DOX-HCl, the intensity mean value was approximately 80% after 24 h incubation, and with the incubation time extended to 48 h, the average fluorescence intensity of HeLa nuclei was higher than 200%. However, even after 48 hours, the average fluorescence intensity of the cells incubated with free DOX remained at 20%. The value obtained for DOX-loaded P3 was between these values for free DOX and DOX-HCl and increased with incubation time, so that after 48 h, the fluorescence intensity increased significantly to 80%. These findings suggested that DOX-loaded polymeric micelles may be suitable for stimulus-responsive nanocarriers.

### *In vitro* cell cytotoxicity

**Fig. 4:**
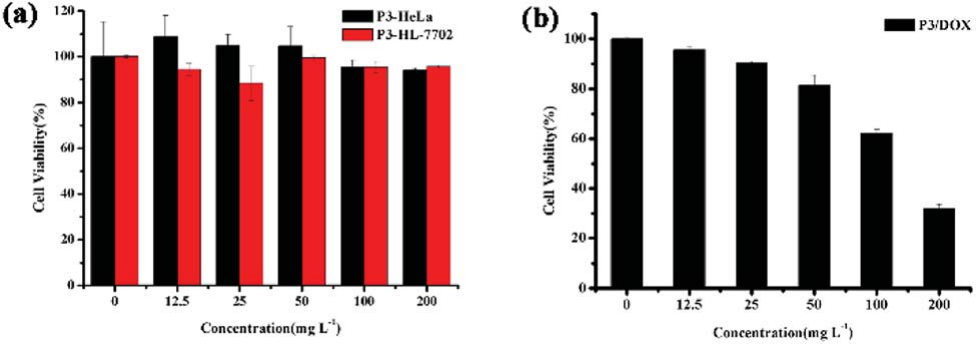
*In vitro* cytotoxicity of (a) P3 in both HeLa and HL-7702 cell; (b) DOX-loaded P3 in HeLa cells.

Cytotoxicity is a critical factor for a carrier, especially for *in vivo* drug delivery. Fig. 4 shows the *in vitro* cytotoxicity results for P3 using the MTS assay method in both HeLa and HL-7702 cells. As shown in Fig. 4(a), all of the concentration of the copolymer exhibited low cytotoxicity towards HeLa and HL-7702 cells. The cell viability was high even at a high polymer concentration (200 mg L^−1^), indicating that the amphiphilic graft copolymer of P3 is highly biocompatible and nontoxic. Furthermore, Fig. 4(b) shows that the *in vitro* cytotoxicity values for cell viability obtained using the MTS assay method in HeLa cells were decreased by DOX-loaded polymeric micelles P3, and the cytotoxicity of DOX-HCl and free DOX was examined by the same method, as shown in Fig. S13.† All of the concentration of the free DOX exhibited low cytotoxicity towards HeLa cells, while the cell viability decreased sharply by 95% for DOX-HCl, while for DOX-loaded polymeric micelles P3, the cell viability decreased sharply by 70% after 48 h of incubation; these results provide further proof that DOX-loaded polymeric micelles P3 have sustained release effect on DOX.

## Conclusions

In summary, we have successfully constructed a novel high-drug-loading, dual stimulus-responsive drug-release system based on a rearrangement of borate ester bond and a hydrolysis of an ester bond. Compared to the common drug control release systems, P3 exhibits high entrapment efficiency and drug loading capacity, and the synergetic characteristics of two stimuli. Such a dramatic loading capacity and synergetic characteristics is anticipated to achieve unprecedented advances in the development of stimuli-sensitive drug-delivery systems that are deemed to be a promising vehicle for drug-delivery nanocarriers.

## Conflicts of interest

There are no conflicts to declare.

## Supplementary Material

RA-009-C8RA09770D-s001
