# Supplementary material for: Synthesis of high drug loading, reactive oxygen species and esterase dual-responsive polymeric micelles for drug delivery
Source: RSC Adv. 2019 Jan 18;9(5):2371–8. doi: 10.1039/c8ra09770d (PMC9059833; doi:10.1039/c8ra09770d)
Supplement: RA-009-C8RA09770D-s001 [file RA-009-C8RA09770D-s001.pdf]

Electronic Supplementary Material (ESI) for RSC Advances.
This journal is © The Royal Society of Chemistry 2019

Supporting Information for

Synthesis of high drug loading, reactive oxygen species and esterase dual-responsive polymeric micelles for drug delivery

Nan Wang,[a] Xiao-Chuan Chen,[b] Ruo-Lin Ding,[c] Xian-Ling Yang,[a] Jun Li,[a] Xiao-Qi Yu,[a]* Kun Li,[a] Xi Wei[b]*

[a] Key Laboratory of Green Chemistry and Technology, Ministry of Education, College of Chemistry, Sichuan University, Chengdu, China 610064 E-mail: xqyu@scu.edu.cn

[b] Operative Dentistry and Endodontics, Guanghua School of Stomatology, Affiliated Stomatological Hospital, Guangdong Province Key Laboratory of Stomatology, Sun Yat-sen University, Guangzhou, Guangdong, China 510055 E-mail: weixi@mail.sysu.edu.cn

[c] West China College of Stomatology, Sichuan University, Chengdu, China 610064

Experimental section:

1. Synthesis routes:

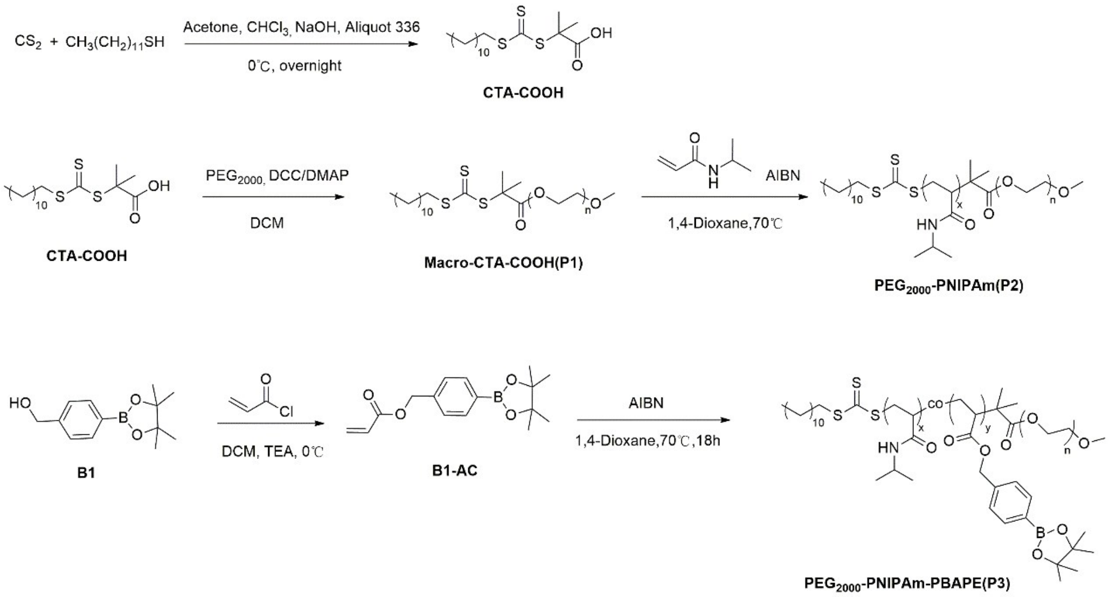

Figure S1. Synthetic route of polymers based on based on esterification and reversible addition - fragmentation chain transfer polymerization.

2. Preparation of polymeric micelles P2

Micelles of **P2** were prepared using the dialysis method. The polymers (30 mg) were dissolved in 6 mL THF. Then, 5 mL deionized water was added to the solution under gentle stirring. After stirring for 2 h at room temperature, the solution was transferred into a dialysis membrane (MWCO 2000 Da) and dialyzed for 48 h against deionized water. The outer phase was replaced with fresh deionized water every 6 h. The resulting solution was finally lyophilized.

2. Preparation of DOX-loaded polymeric micelles **P2**

Here, we identified a common anticancer drug, doxorubicin (DOX) as a model drug, and the water-insoluble DOX was encapsulated into **P2** micelles by adding 3 mL of 1 mg mL^{-1} DOX in THF to 30 mg **P2**, followed by adding 12mL DI-water at a very low speed. And then the solution was stirring at room temperature for twelve hours in absolute darkness condition. Then, the solution was filtered through a 0.45 μm

syringe filter.

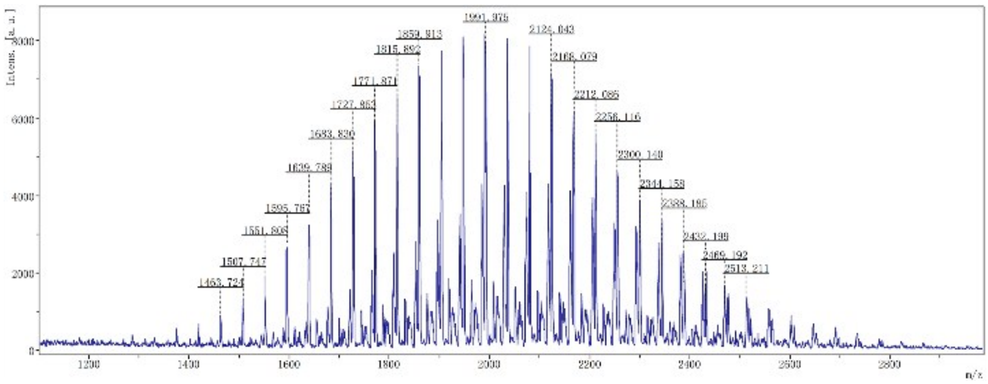

Figure. S2. The MALDI−TOF spectrum of **P1**.

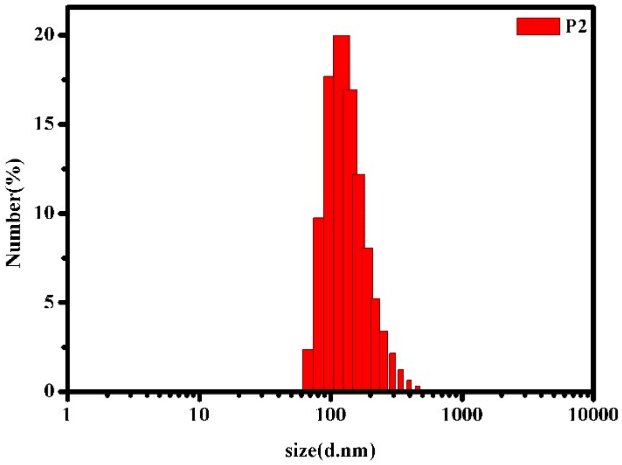

Figure. S3. Particle size distributions of **P2** at 25℃ (pH = 7.40).

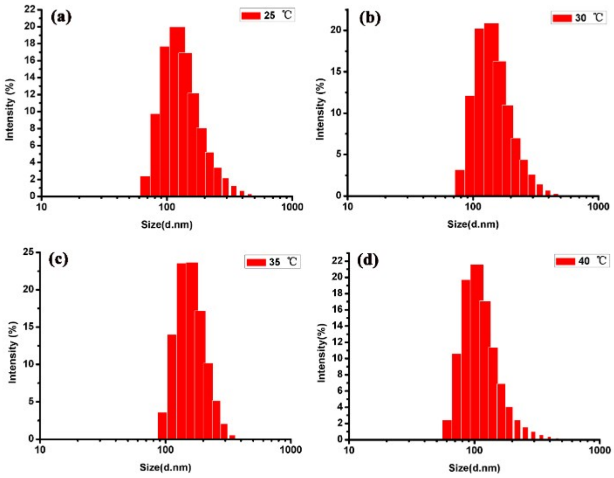

Figure. S4 . The size distributions of **P2** at different temperature. (a-d):25℃, 30℃,

35°C, and 40°C.

P2 is a monoblock copolymer of isopropyl acrylamide. As we can see from the Figure. S4, with the increase of temperature, the particle size distribution of **P2** decreases. The particle size shrinks from 122 nm to 105 nm. This may be due to the shrinkage of the chain segment caused by the temperature-sensitive properties of **P2.**

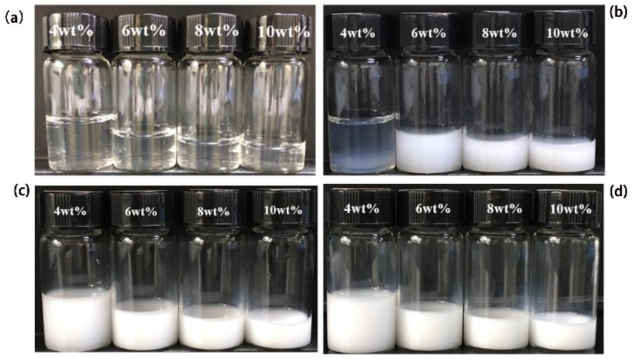

Figure. S5. The **P2** solutions at different temperatures and different concentrations: (a-d) 25℃, 30℃, 35℃ and 40℃.

In order to further prove the temperature-sensitive properties of **P2** macroscopically, we prepared **P2** solutions at different concentrations. At 25℃, all solutions are clear, as the temperature rises, the solution becomes cloudy, and the lower the **P2** concentration, the narrower the range of change. This naked-eye turbidity is largely caused by the contraction of **P2** segments.

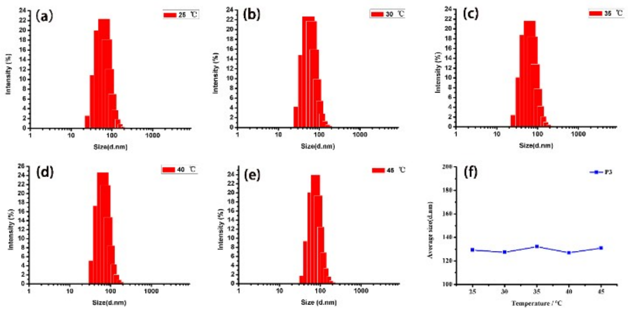

Figure. S6. (a-e). The size distributions of **P3** at different temperature. (f) The temperature-dependence curves of time.

We also investigated temperature-sensitive properties of **P3**, with the increase of temperature, the particle size of **P3** remained very uniform, and the size is around 130 nm.

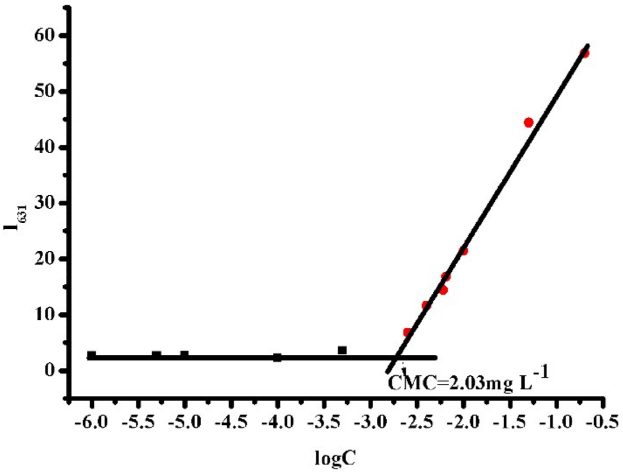

Figure. S7. Critical micelle concentration determination of **P3**. Fluorescence intensity at λem = 631 nm of Nile red as function of logarithm of the polymer concentrations in PBS buffer solution (pH = 7.40).

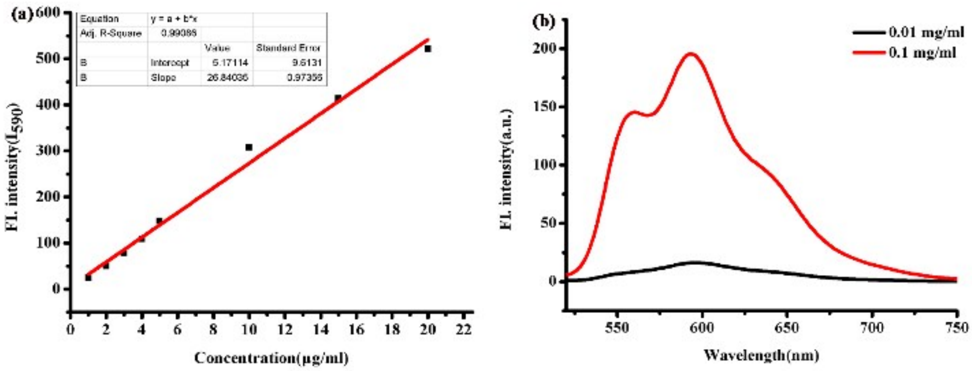

Figure. S8. The standard curve for determination of free DOX content in 90% DMF aqueous solution (a) and (b) the emission of **DOX-loaded P3** in 90% DMF aqueous solution with different concentrations; λem = 590 nm.

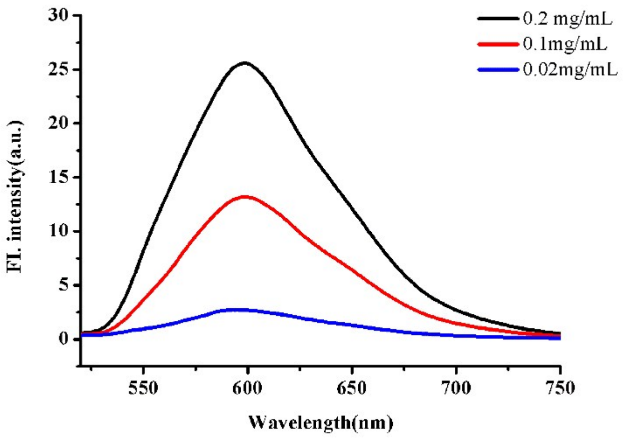

Figure. S9. The emission of **DOX-loaded P2** in 90% DMF aqueous solution with different concentrations; λem = 590 nm.

The standard curve for the determination of the free DOX content was measured by the fluorescence method with a Hitachi F-7000 spectrometer. The concentration of free DOX was calculated by the standard curve, as shown in Figure S8. The encapsulation efficiency and loading of free DOX were obtained by the following formulas:

LC(%) = (Weight of loaded drug)/(Weight of drug loaded micelle) ×100% (1)

EE(%) = (Weight of loaded drug)/(Weight of drug in feed)×100% (2)

The entrapment efficiency and loading capacity of free DOX were calculated by the standard curve, according to the following equation. Entrapment efficiency = 3.95%; loading capacity = 0.36 wt%. According to the calculation results, we can clearly see that the LC and EE of compound **P2** is very low when compared with **P3**, Therefore, most of the LC and EE value are due to the introduction of PBAPE group.

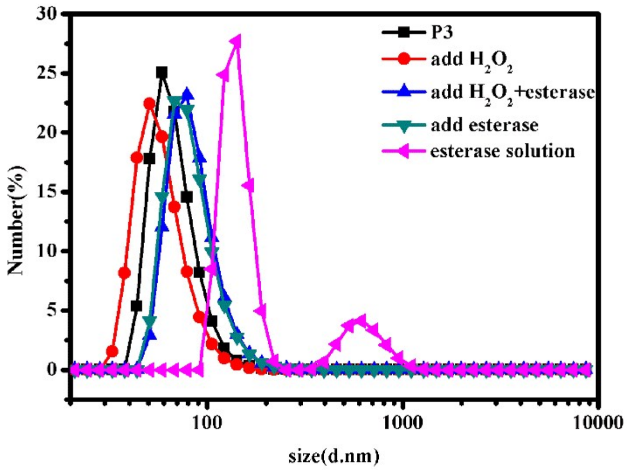

Figure. S10. Particle size distributions of **P3** under different conditions but coincide with the conditions released in vitro. (Black : only P3 polymeric micelles; Red : 0.2mg mL^{-1} **P3** polymeric micelles add 10 mM H_2O_2 solution; Blue : 0.2 mg mL^{-1} **P3** polymeric micelles add 10 mM H_2O_2 solution and 30 μg mL^{-1} esterase; Green : 0.2 mg mL^{-1} **P3** polymeric micelles 30 μg mL^{-1} esterase; Purple : 30 μg mL^{-1} esterase solution.)

Table S1 Particle size distributions of P3 under different conditions

Sample	**Black**	**Red**	**Blue**	**Green**	**Purple**
Size^a(nm)	64.84 ± 0.22	59.53 ± 0.64	83.05 ± 0.63	84.64 ± 0.26	576.1 ± 21.2
PDI^a	0.154 ± 0.001	0.149 ± 0.02	0.153 ± 0.05	0.17 ± 0.01	0.578 ± 0.045

a: The size and PdI (polydispersity index) of the micelles were determined by DLS.

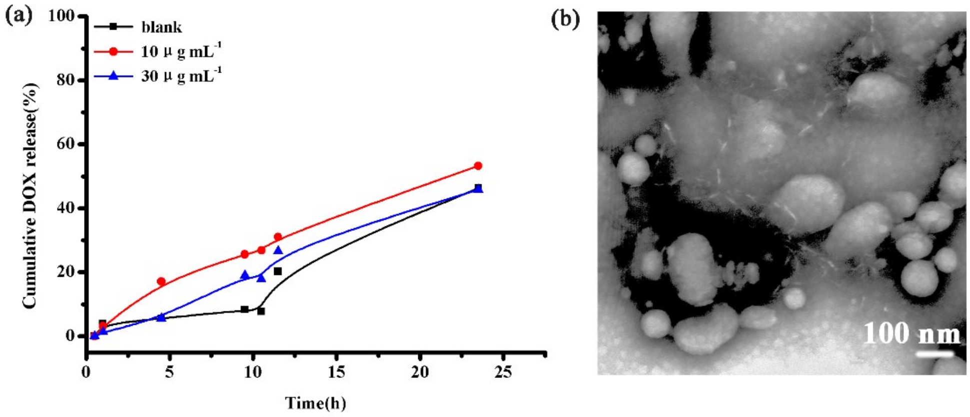

Figure. S11. (a) Release profiles of DOX from 0.2 mg mL^{-1} P3 micelles at different conditions (Black : blank; red : 10 μg mL^{-1} esterase; Blue: 30 μg mL^{-1} esterase); (b) TEM image of DOX-loaded P3 with 30 μg mL^{-1} esterase.

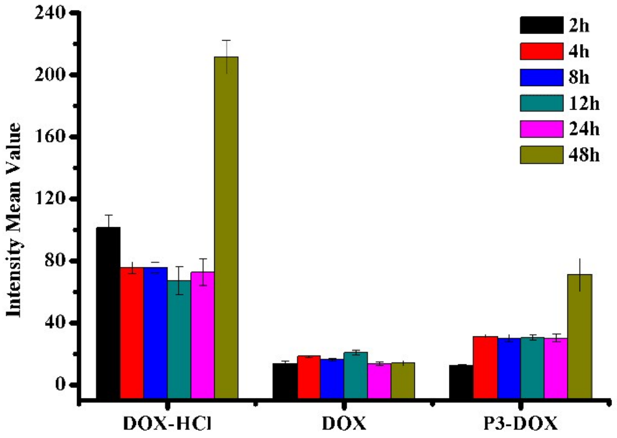

Figure. S12. Intensity mean value in the nucleus of HeLa cell at different times.

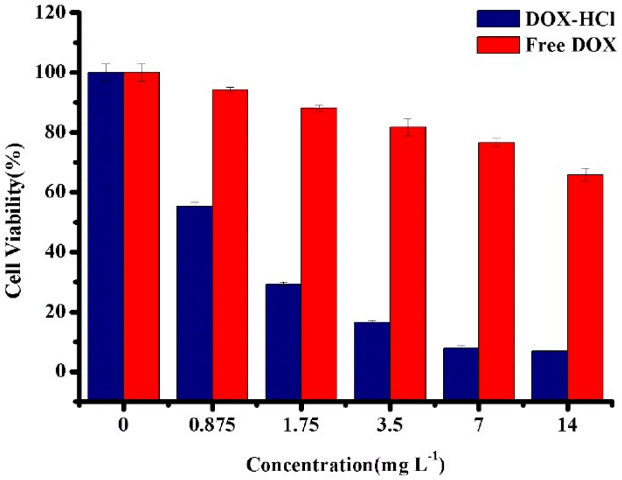

Figure. S13. In vitro cytotoxicity of DOX-HCl and free DOX in HeLa cells**.**

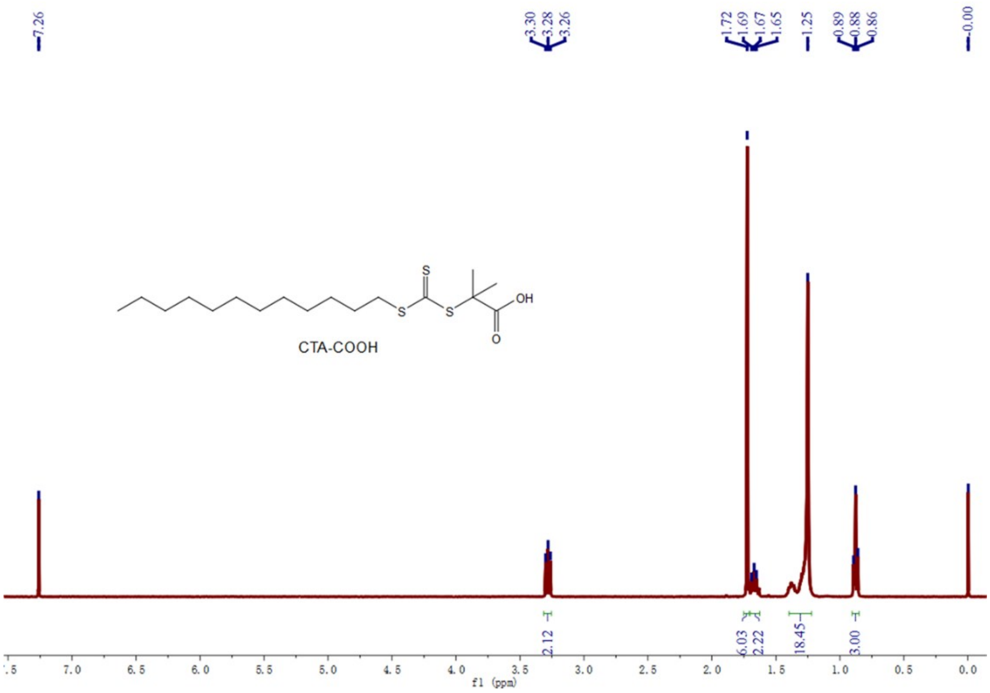

Figure. S14. ^{1}H NMR spectrum of **CTA-COOH**.

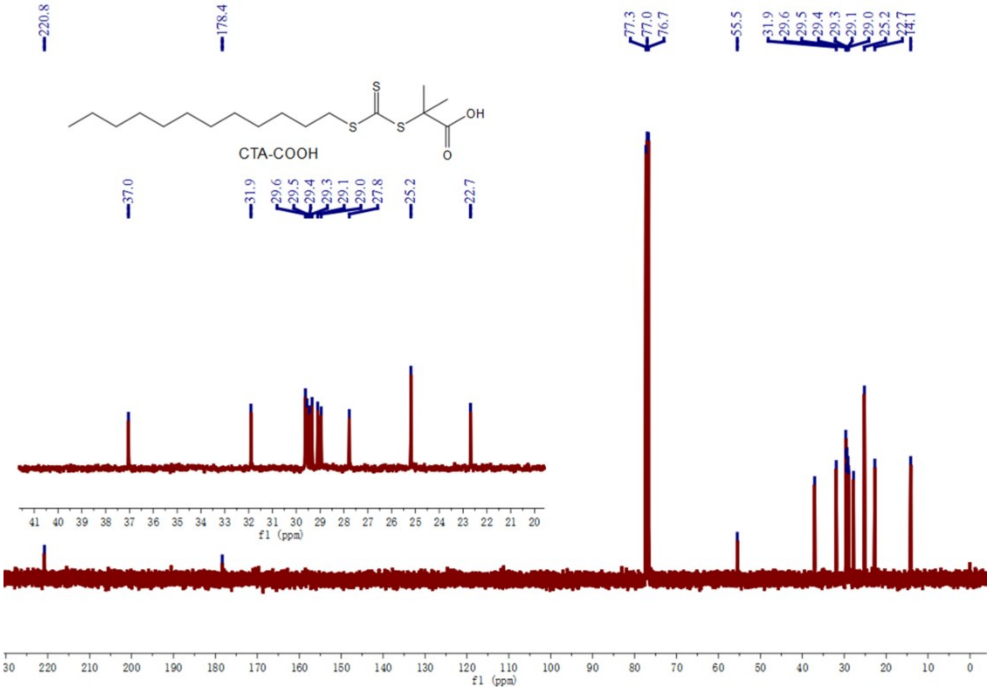

Figure. S15. ^{13}C NMR spectrum of **CTA-COOH**.

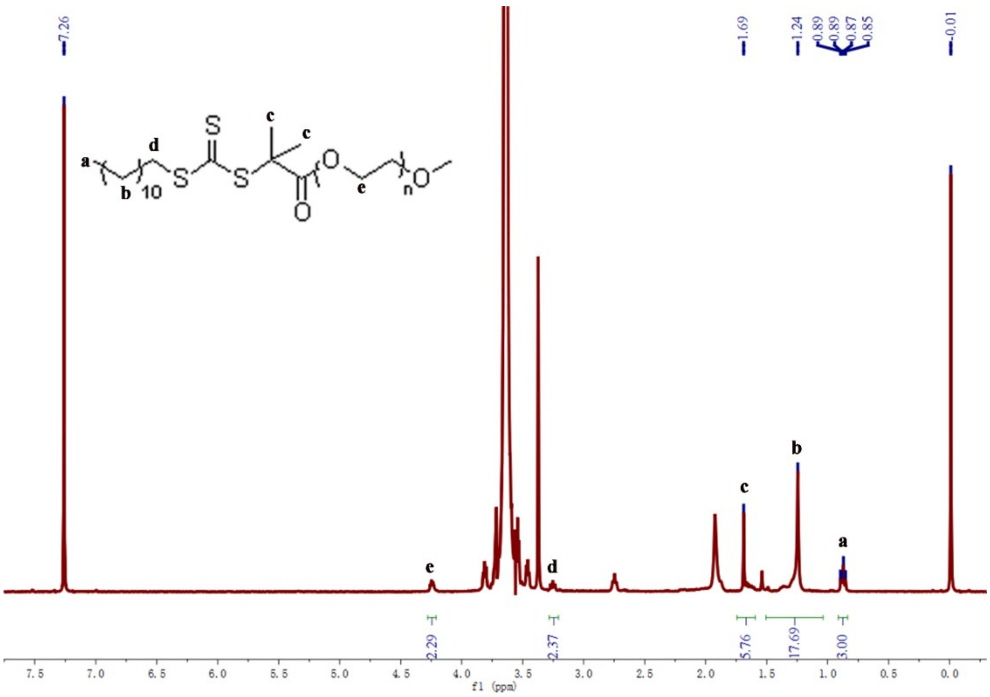

Figure. S16. ^{1}H NMR spectrum of **macro-CTA-COOH(P1)**.

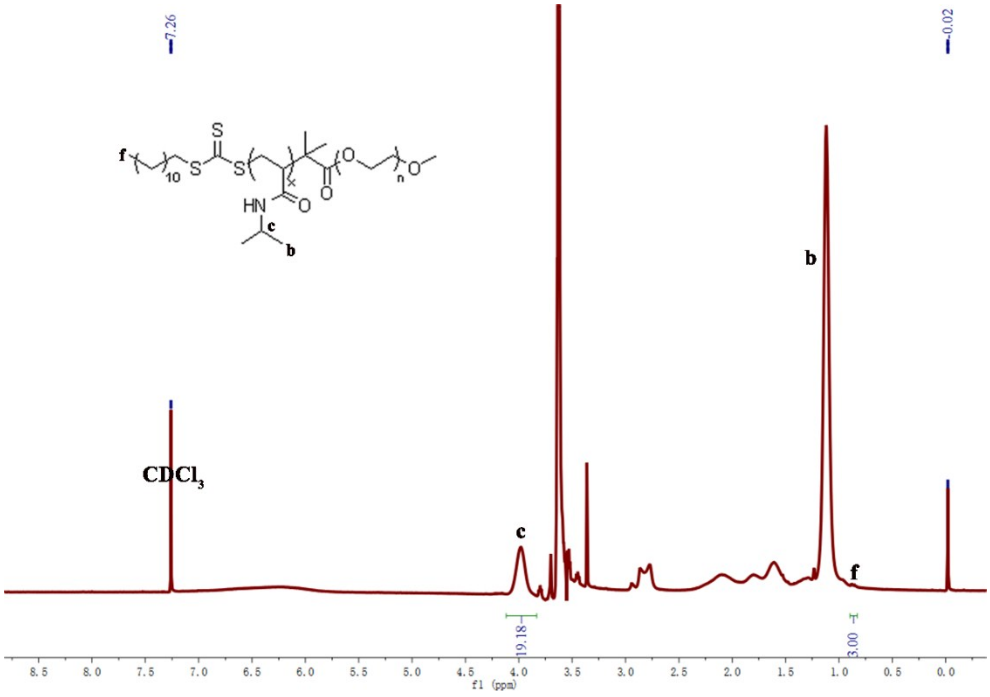

Figure. S17. ^{1}H NMR spectrum of **mPEG$_{2000}$-PNIPAM(P2)**.

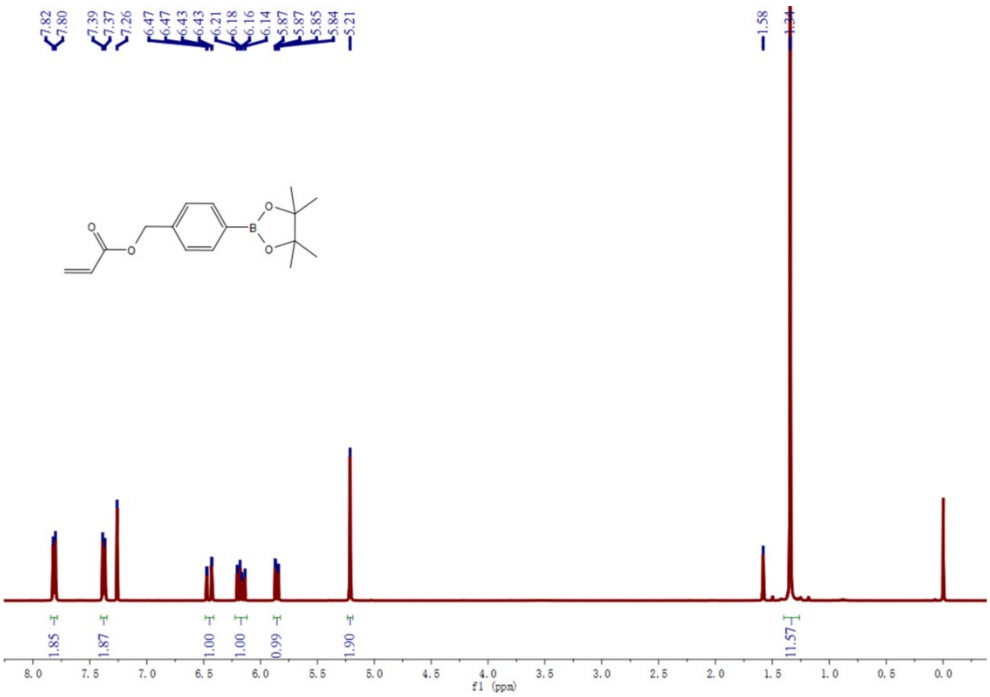

Figure. S18. ^{1}H NMR spectrum of **B1-AC**.

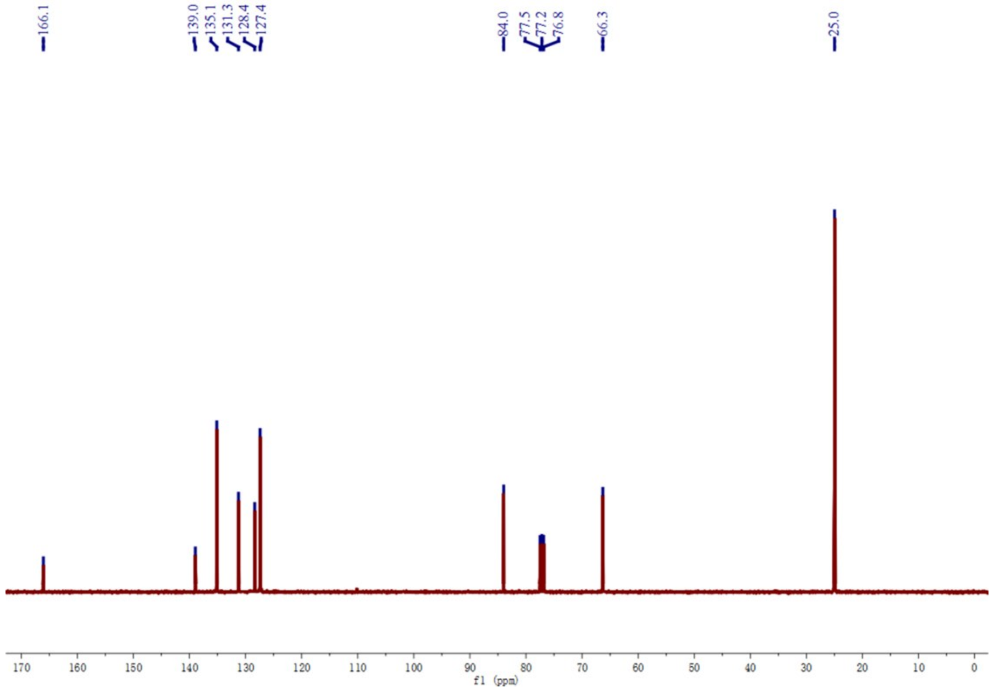

Figure. S19. ^{13}C NMR spectrum of **B1-AC**.

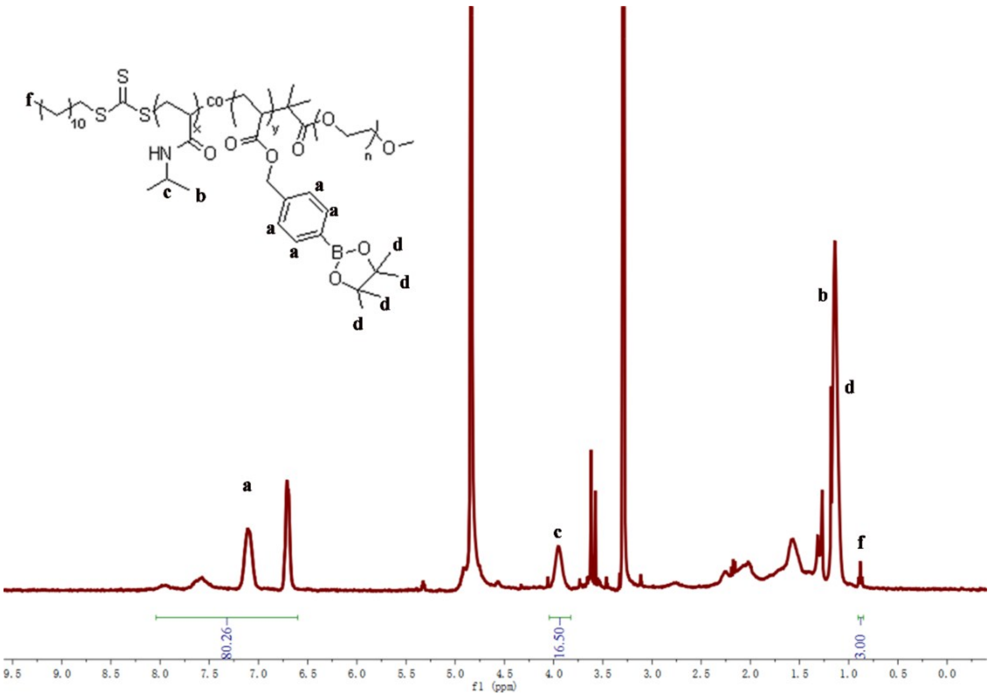

Figure. S20. ^{1}H NMR spectrum of **mPEG$_{2000}$-PNIPAM-co-PBAPE(P3)**.

According to the results of NMR, the degree of polymerization of NIPAM and BAPE can be calculated. The degree of polymerization of NIPAM is 7, and the degree of polymerization of BAPE is 7 too, it is shown that the two segments are proportionally aggregated at a ratio of 1:1.

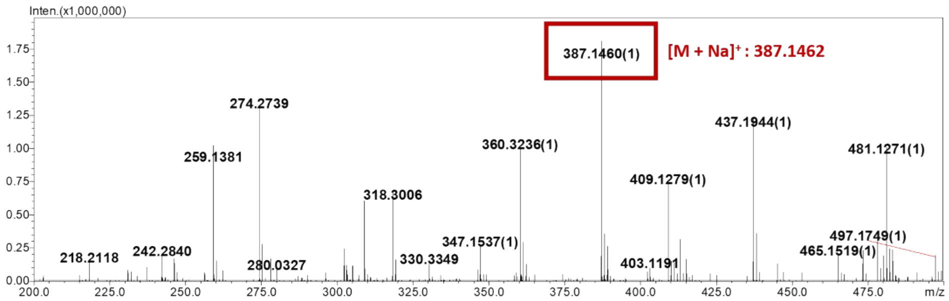

Figure. S21 ESI-MS Data of **CTA-COOH**.

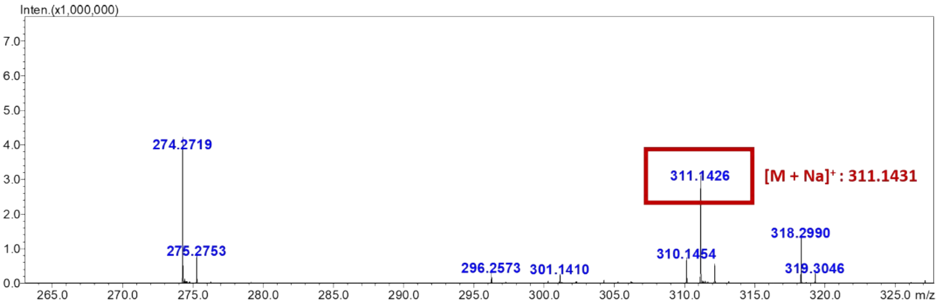

Figure. S22 ESI-MS Data of **B1-AC**.
